# Passive Immunoprophylaxis against Respiratory Syncytial Virus in Children: Where Are We Now?

**DOI:** 10.3390/ijms22073703

**Published:** 2021-04-02

**Authors:** Alessandro Rocca, Carlotta Biagi, Sara Scarpini, Arianna Dondi, Silvia Vandini, Luca Pierantoni, Marcello Lanari

**Affiliations:** 1 Pediatric Emergency Unit, Scientific Institute for Research and Healthcare (IRCCS), Sant’Orsola Hospital, 40138 Bologna, Italy; alessandro.rocca4@unibo.it (A.R.); carlotta.biagi@aosp.bo.it (C.B.); arianna.dondi@aosp.bo.it (A.D.); luca.pierantoni@aosp.bo.it (L.P.); marcello.lanari@unibo.it (M.L.); 2 Specialty School of Paediatrics—Alma Mater Studiorum, University of Bologna, 40126 Bologna, Italy; 3 Pediatrics and Neonatology Unit, Imola Hospital, 40026 Imola, Italy; s.vandini@ausl.imola.bo.it

**Keywords:** respiratory syncytial virus, monoclonal antibodies, palivizumab, children

## Abstract

Respiratory syncytial virus (RSV) represents the main cause of acute respiratory tract infections in children worldwide and is the leading cause of hospitalization in infants. RSV infection is a self-limiting condition and does not require antibiotics. However hospitalized infants with clinical bronchiolitis often receive antibiotics for fear of bacteria coinfection, especially when chest radiography is performed due to similar radiographic appearance of infiltrate and atelectasis. This may lead to unnecessary antibiotic prescription, additional cost, and increased risk of development of resistance. Despite the considerable burden of RSV bronchiolitis, to date, only symptomatic treatment is available, and there are no commercially available vaccines. The only licensed passive immunoprophylaxis is palivizumab. The high cost of this monoclonal antibody (mAb) has led to limiting its prescription only for high-risk children: infants with chronic lung disease, congenital heart disease, neuromuscular disorders, immunodeficiencies, and extreme preterm birth. Nevertheless, it has been shown that the majority of hospitalized RSV-infected children do not fully meet the criteria for immune prophylaxis. While waiting for an effective vaccine, passive immune prophylaxis in children is mandatory. There are a growing number of RSV passive immunization candidates under development intended for RSV prevention in all infants. In this review, we describe the state-of-the-art of palivizumab’s usage and summarize the clinical and preclinical trials regarding the development of mAbs with a better cost-effectiveness ratio.

## 1. Introduction

### 1.1. Virology and Pathogenesis of RSV

**Figure 1:**
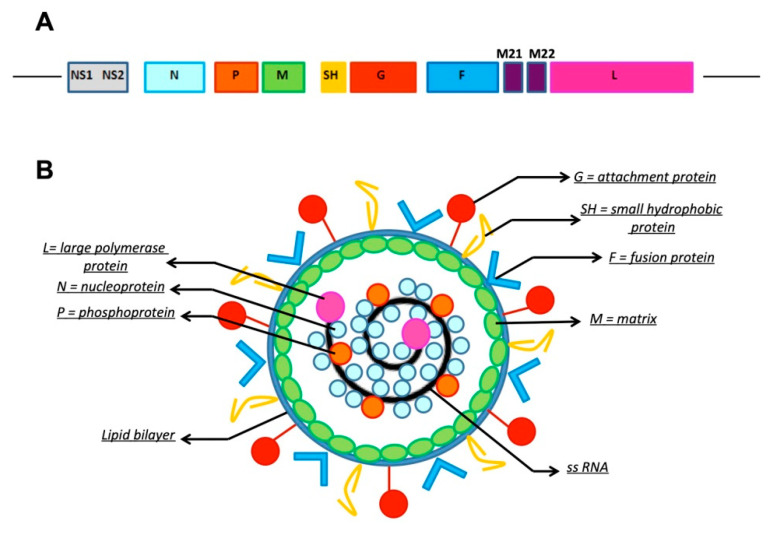
Structure of respiratory syncytial virus (RSV). (**A**). Genomic RNA. L: large polymerase protein; M: matrix; M2.1, M2.2: regulatory subunits; NS1, NS2: non-structural protein 1, 2; N: nucleoprotein; P: phosphoprotein; SH: small hydrophobic protein; G: attachment protein; F: fusion protein. (**B**). RSV virion structure.

Respiratory syncytial virus (RSV) is a non-segmented, single-stranded, negative-sense RNA virus belonging to Genus *Pneumovirus*, Subfamily *Pneumovirinae*, Family *Paramyxoviridae*, Order *Mononegavirales* [1]. RSV encodes 11 proteins, of which the proteins of the lipid envelope are involved in the mechanisms of viral attachment and, subsequently, in the infection and development of the respiratory disease (Figure 1). Four proteins are associated with the lipid double layer: the matrix (M) protein, the small hydrophobic (SH) protein, and the two glycosylated surface proteins: F (Fusion) and G (attachment glycoprotein). The virus exists worldwide in two antigenic subgroups, A and B, as well as multiple genotypes, which can co-circulate during an epidemic season [2,3]. The antigenic variability between RSV A and B is due to variations in the G protein, while the F protein exhibits relative stability, making it a major target for vaccine and monoclonal antibody (mAb) development. The pathogenesis of RSV infection is complex and variable. The tropism of the virus is high for epithelial respiratory cells. Histopathological findings include necrosis of respiratory tract cells, proliferation of the bronchiolar epithelium, and infiltration of monocytes, T-cells, and neutrophils between vessels and small airways [4]. The G and F proteins are mostly involved in the pathogenesis of the infection since the G protein mediates the adhesion to respiratory tract cells, while the F protein is responsible for the entry of the virus into the cells and in the insertion of viral RNA in the cell, which is responsible for the formation of syncytia [5]. Two different mechanisms are involved in the development of airway inflammation: the necrosis of airway epithelial cells subsequent to the cytopathological effect of RSV and the immune response to RSV, resulting in inflammation and subsequent destruction. Innate immunity is firstly involved against virus infection, before induction of the adaptive immune response [3].

### 1.2. The Burden of RSV Disease in Children

RSV represents the main cause of acute lower respiratory tract infections (LRTI) in children worldwide. It is responsible for more than 30 million pediatric LRTI and more than 50,000 in-hospital deaths per year worldwide, with a subsequent great requirement of healthcare resources, hospitalizations, and intensive care admissions [6,7]. About 45% of hospitalizations and in-hospital deaths occurred in infants younger than six months [6]. The clinical course of RSV infection can consist of a wide range of acute upper and lower respiratory tract infections, from mild rhinitis at one extreme to severe bronchiolitis and respiratory failure at the other [1]. According to the American Academy of Pediatrics (AAP) [8], bronchiolitis is a viral LRTI involving children younger than two years characterized by a set of symptoms including increased respiratory effort, tachypnea, and wheezing and/or crackles on chest auscultation, which follow a few days of rhinorrhea, cough and, occasionally, fever. RSV bronchiolitis symptoms peak around day five of the illness and in most cases improve by day 10. Indicators for hospital admission are respiratory rate over 60 breaths/minute, marked chest wall retractions, peripheral oxygen saturation (SpO_2_) lower than 92%, central cyanosis, apnea, and poor oral fluid intake due to breathlessness [9]. Moreover, infants with RSV bronchiolitis were at higher risk for developing asthma and recurrent wheezing [10]. The diagnosis of bronchiolitis is clinical, and most children manifest a mild condition and can be managed at home. Hospitalization is required in 3% of all cases, of which 2–6% need pediatric intensive care [11]. RSV is estimated to cause up to 90% of pediatric bronchiolitis hospitalizations [12]. High-risk children are infants with chronic lung disease (bronchopulmonary dysplasia, BPD), congenital heart disease (CHD), neuromuscular disorders, immunodeficiencies, and extreme preterm birth [13,14].

Treatment of RSV bronchiolitis is primarily supportive, including the use of supplemental oxygen in case of desaturation (SpO_2_ below 92%), fluid replacement therapy, and decongestant nose drops [15]. The only antiviral drug currently approved is ribavirin; however, its use is limited due to its potential toxicity [16]. Since RSV bronchiolitis is a viral illness, antibiotics do not alter the course of disease. The use of antibiotics should be reserved for cases in which the disease is severe enough to require admission into the Pediatric Intensive Care Unit (PICU) or in the case of positive cultures or molecular tests showing the presence of secondary bacterial infection [17,18]. Infants with bronchiolitis requiring mechanical ventilation have been reported to have high rates of bacterial coinfection (21–26%), which warrant antibiotic use in these patients [19,20]. Apart from these patients, the risk of bacteremia is lower in children with bronchiolitis and fever (0.2%) compared to those with fever with no recognizable disease (2–7%) [21]. However, the young age of these patients and the presence of fever frequently raise doubts about undetected bacterial coinfection, leading clinicians to prescribe unnecessary antibiotics in up to 85% of cases [22,23,24]. Antibiotic therapy is often prescribed in children undergoing chest x-ray (CXR) owing to similar radiographic appearance of infiltrate and atelectasis [25]. Antibiotics need to be used cautiously due to their potential side effects, increased costs, and contribution to the emergence of bacterial resistance, an increasing issue. For these reasons, in the last years, many quality improvement methodologies have been attempted to minimize the use of CXR in these types of patients [26,27]. However, no significant enhancement into clinical practice has been reached until now. 

Prevention plays an essential role in reducing the burden of RSV disease in children and avoiding inappropriate therapies, including antibiotics. Although many vaccine candidates have been in clinical evaluation in the last few decades, none, to date, has reached licensing [28]. Therefore, while waiting for an effective vaccine, passive immune prophylaxis in children should be mandatory. To date, two prophylaxis products have been considered to prevent RSV infections: first polyclonal intravenous immunoglobulin and later intramuscular mAbs. The only licensed passive immunoprophylaxis nowadays is palivizumab, a humanized murine mAb whose prescription is restricted to high-risk children due to its high cost [29]. Nevertheless, many hospitalized RSV-infected children do not fully meet the criteria for immune prophylaxis. Thus, there are a growing number of RSV passive immunization candidates under development intended for RSV prevention in all infants with a better cost-effectiveness ratio than palivizumab. 

In the following paragraphs, we retrace the history of the passive RSV prophylaxis, describe the state-of-the-art of the use of palivizumab, and summarize the clinical and preclinical trials regarding the development of new mAbs.

## 2. Passive Prophylaxis against RSV

### 2.1. RSV Immune Globulin Intravenous

The use of RSV immune globulin intravenous (RSV-IGIV; RespiGam^®^, Massachusetts Public Health Biologic Laboratories, and MedImmune, Inc, Gaithersburg, MD, USA) was one of the first approaches tested to prevent RSV infection. RSV-IGIV is made up of a pool of polyclonal antibodies derived from the plasma of donors with naturally high circulating levels of RSV-neutralizing antibodies [30]. In 1993, Groothuis et al. evaluated the effect of these antibodies in 249 children with BPD, CHD, and in preterm infants ≤35 weeks of gestational age (wGA), reporting a decrease in the length of hospital stay and in symptom duration by administering a high dose (750 mg/kg) of RSV-IGIV for five times during the RSV season [31]. However, six children died during the trial, five of them being affected by CHD. Thus, two subsequent studies were carried out to clarify the safety and efficacy of RSV-IGIV: the PREVENT trial [32] and the CARDIAC trial [33]. The PREVENT study in 1997 demonstrated that monthly administration of RSV-IGIV was safe, well-tolerated and effective in reducing the incidence of hospitalization in infants with a history of prematurity (≤35 wGA) and in children with BPD aged less than two years [32]. The CARDIAC trial [33] showed that RSV-IGIV did not reduce hospitalization in all children with CHD, but only in infants younger than 6 months of age. Moreover, during the study, there was a higher frequency of cyanotic episodes and poor outcome after surgery among children with cyanotic CHD in the RSV-IGIV group than in the control group, probably due to hyperviscosity. Taking into account all these data, RSV-IGIV has not been approved for use in children with CHD [34]. In 1996, RespiGam^®^ was therefore licensed by the Food and Drug Administration (FDA) for use in premature infants and children with BPD, thus qualifying a large number of infants for this medication [35]. Even with the positive outcomes in this population, RespiGam^®^ was characterized by several weaknesses: fluid overload, hypoxemia or cyanosis, adverse events in children with CHD, intravenous administration, and the need to delay vaccination with live vaccines. Thus, this product was voluntarily withdrawn from the market in 2004, following the licensing of the first anti-RSV mAb, Palivizumab [36]. RI-001 (ADMA Biologicals) is another intravenous immunoglobulin preparation obtained from pooled plasma from donors with high titers of RSV. A Phase-II clinical trial, conducted enrolling immunocompromised RSV-infected patients between 2 and 65 years of age, showed a statistically significant increase in anti-RSV-neutralizing antibody [37]. In another study, RI-001 was administered for compassionate use to 15 patients with documented RSV-LRTI who had failed conventional therapy. All patients who received RI-001 within four days after the diagnosis of RSV infection survived; serum samples showed a four-fold or greater rise in RSV antibody titers from baseline. The drug was well-tolerated, and there were no reports of serious adverse events [38]. Subsequently, RI-002 was created adding polyclonal antibodies against Streptococcus pneumoniae and Haemophilus influenzae type B to RI-001 formulation. A phase-III trial studied the prevention of bacterial infections in patients with primary immunodeficiencies, but it did not report the role of RI-002 in preventing RSV infections [39].

### 2.2. Monoclonal Antibodies 

In the 1990s, the development of humanized mAbs against the RSV surface glycoproteins started, with the aim to obtain prophylaxis with higher specificity and improved potency compared to RSV-IGIV. This new approach presents many advantages. Being mAbs therapeutic doses contained in low volumes, their administration reduces the risk of fluid overload compared to RSV-IGIV. Moreover, since every product is composed of a unique type of antibody (anti-RSV), their administration has no effect on subsequent vaccine schedules. Lastly, mAbs are safer than RSV-IGIV considering iatrogenic blood-borne pathogen transmission [36]. In the 1990s, the first three mAbs (HNK20, SB209763, and MEDI-493/palivizumab) were studied in clinical trials. All these mAbs were antibodies against the RSV F glycoprotein. The F-protein was chosen in order to ensure both the A and B VRS subtype neutralization, preventing cellular infection by avoiding fusion between the viral membrane and the cell membrane, and the formation of syncytia in the lung, by blocking cell-to-cell spread of the virus [34]. HNK20 was an Immunoglobulin (Ig) A mAb obtained by fusion of myeloma cells with lung lymphocytes from the RSV-immunized mouse model. Initial studies with intranasal administration to mice and monkeys gave hopeful results about protection against both upper respiratory tract infections and LRTI caused by RSV [40,41]. Nevertheless, after the process of humanization, the product significantly lost its antiviral activity both in vitro and in vivo with animal models, and its research was abandoned [42]. SB209763 (also known as RSHZ19) was a reshaped human IgG_1_ mAb. After its promising results in neutralizing RSV in cotton rats and healthy volunteers, SB209763/RSHZ19 showed lower clinical efficacy when it was directly compared to another IgG_1_ mAb, MEDI-493 (also known as palivizumab) in infants at risk for severe RSV disease. These results led the FDA to approve palivizumab alone for passive immunization against RSV in high-risk children [36,43,44,45,46].

#### 2.2.1. Palivizumab

Palivizumab (MEDI-493, Synagis^®^) is a humanized IgG_1_ monoclonal antibody to the RSV F-protein, developed over 10 years by MedImmune Inc. (Gaithersburg, MD, USA) [47]. It was approved for the first time by FDA in 1998 for RSV prophylaxis of high-risk children, while the European Agency for the Evaluation of Medicinal Products (EMEA) approved it the following year [48]. Phase III/IV studies [29,49] have proven the efficacy of palivizumab in reducing RSV hospitalization, number of days spent in hospital, incidence of PICU admission and severity of LRTI, in children with prematurity, BPD, and CHD, who received five doses of palivizumab (15 mg/kg) by intramuscular injection every 30 days, as recommended by the Phase I/II trials [50,51]. In particular, the IMpact-RSV Study Group conducted a randomized, double-blind, placebo-controlled trial involving 139 centers in the United States, the United Kingdom, and Canada during the 1996 and 1997 RSV season. The study enrolled premature infants (<35 wGA) under 6 months of age at the beginning of the epidemic season and premature children with BDP <2 years of age, and in need of medical therapy in the six months before the beginning of the epidemic season [29]. Prophylaxis with palivizumab resulted in a 55% reduction in RSV hospitalization. Moreover, the palivizumab group had a shorter in-hospital stay and a lower incidence of PICU admission compared to the placebo group. It is on the basis of this last study that, in 1998, the AAP developed the first guidelines on palivizumab [52]. These guidelines recommended the administration of palivizumab in the same populations of children recruited in the IMpact-RSV Study, except for infants 33 to 35 wGA, for whom the risk of hospitalization for severe respiratory illness was considered low, and the cost and logistical difficulties associated with palivizumab administration were assessed to be higher than the potential benefits. Thus, for this group of infants, additional risk factors were required for receiving prophylaxis: neurologic disease, presence of young siblings, child care attendance, passive tobacco smoke exposure, planned cardiac surgery, or difficulties regarding medical support for severe respiratory disease. In 2003 the AAP provided additional recommendations for infants and children with CHD [53]. They included children under two years of age with hemodynamically significant CHD among patients who deserve to receive palivizumab, especially those who receive medication to control congestive heart failure, those with moderate to severe pulmonary hypertension, and those with cyanotic CHD. In 2009 there was an update of the AAP guidelines: risk factors for severe disease among infants born between 32 and 35 wGA have been modified to include only childcare attendance or living with other children younger than five years; a maximum of 3 doses of palivizumab were suggested for this group of infants [54]. The AAP recommendations changed again in 2014 because of the publication of several studies [55,56,57], which demonstrated the greatest increase in the risk for RSV hospitalization in infants born before 29 wGA, with hospitalization rates two to four times higher than later preterm children. According to these results, the AAP guidelines published in 2014 [13], which remained unchanged in 2017 [58] recommended that palivizumab might be given to preterm infants born before 29 wGA at a maximum of five doses during one season. No recommendations for healthy preterm infants of 29 to 35 wGA were provided. Indications for infants with BPD and CHD didn’t change. Unclear evidence has been found for the use of palivizumab in infants with anatomic pulmonary abnormalities or neuromuscular disease, Down syndrome, cystic fibrosis, or immunodeficiency. The 2009 and 2014 AAP guidelines on palivizumab prophylaxis are summarized in Table 1.

Concerning the use of palivizumab in children with CHD, subsequent recommendations have been published in 2017 by an international group of clinicians with expertise in this field [59]. Prophylaxis was recommended for children younger than 2 years with unoperated hemodynamically significant CHD, who are cyanotic, who have pulmonary hypertension, or symptomatic airway abnormalities; for children less than 1 year old with cardiomyopathies requiring treatment; with surgically operated CHD and hemodynamically significant residual problems, or those aged 1–2 years up to 6 months postoperatively; and in children on heart transplant waiting lists or in their first year after heart transplant. 

Given the high cost of palivizumab, cost-effectiveness of this mAb was tested by a large number of cost-benefit analysis, but results were inconsistent, varying considerably across studies, depending on many variables included in calculation model and parameters taken into account [60,61]. For example, there is increasing evidence that RSV infection in premature children may influence long-term respiratory function [62,63,64,65,66]. In 2020 Shi et al. published a systematic review of 41 studies and subsequent meta-analysis, confirming a considerable association between early RSV infection and the development of childhood recurrent wheeze and of asthma at follow-up [67]. Thus, the impact of RSV disease reveals to have short- and long-term consequences and social implications that are difficult to calculate, which are often not included in cost-effectiveness analyses [62,68]. A recent study conducted in the UK showed that palivizumab prophylaxis is cost-effective in preventing severe RSV LRTI in a wider population than currently recommended in guidelines [69]. Narayan et al. have found that palivizumab is cost-effective in premature infants born before 35 wGA without CHD or BPD aged <6 months at the start of the RSV season and in premature infants with CHD or BPD aged <2 years, if a mean of 3.7 doses rather than the five doses, is used. 

As the evidence behind the APP recommendations published in 2014 is not always clear, many countries use earlier guidelines to guide palivizumab prophylaxis [70,71]. In Italy, palivizumab was administered to all preterm infants born before 32 wGA and to those born at 33–35 wGA with certain additional risk factors up to 2016. Driven primarily by cost–benefit consideration, in September 2016, the Italian Drug Agency (AIFA) decided that the financial coverage of palivizumab by the National Health Service in the group of healthy preterms should be limited to infants born before 29 wGA and younger than 12 months at the beginning of the RSV epidemic season [72]. After implementation of these restrictions, several studies were carried out [73,74,75]. In November 2017, in consideration of the new clinical data, the AIFA re-extended the prophylaxis reimbursement to preterm infants born after 29 wGA and younger than 6 months at the beginning of the season [76]. A systematic review of seven Italian reports compared RSV-related hospitalizations during the 2016–2017 season with the hospitalizations of 2 seasons before (2014–2015 and 2015–2016) and one season after (2017–2018) the AIFA limitation. During the 2016–2017 RSV epidemic season, the study showed a higher incidence of RSV bronchiolitis and increased impairment of respiratory function. They also found a higher incidence of hospitalizations and admissions to the PICU, longer hospital stays, and an increase in the number of RSV bronchiolitis in infants born at term, probably because the decreased prophylaxis in preterms may have caused a wider infection diffusion in all the groups of infants. 

Multiple studies have been made worldwide to understand the impact of the change of 2014 AAP guidelines on hospitalization risk and rates, severity, and cost in preterm infants born 29–34 wGA [77,78]. In 2020, Krilov et al. published a review collecting and evaluating several of these works [77]: they found a substantial reduction in palivizumab use after 2014, in association with an increased risk for RSV hospitalization in 29–34 wGA infants compared to term infants, and with higher severity and healthcare utilization. Taking into account the proven usefulness of palivizumab and its high cost, identification, and prediction of risk factors could help to choose infants who are at risk and to employ a cost-effective use of palivizumab, until new methods of prevention become available [79,80,81,82]. Blanken et al. [80] developed a risk scoring tool that predicts RSV hospitalization in moderate-late preterm infants. The best predictors identified were proximity of birth to the RSV season, second-hand smoke exposure, and the presence of siblings and/or daycare attendance. Young chronological age during the RSV season, having school-age siblings, daycare attendance, breastfeeding less than 2 months and small for gestational age were identified as risk factors for hospitalization by a systematic review published by Mauskopfare et al. [82].

#### 2.2.2. Latest Monoclonal Antibodies against RSV

Over the last decades, efforts continued to develop new mAbs with a potential better cost-effectiveness ratio compared to palivizumab, or with extended serum half-life [30,36]. RSV mAbs in clinical development are summarized in Table 2. 

The MEDI-524 mAb (also known as motavizumab), another IgG_1_ anti-RSV F glycoprotein, was created remodeling the heavy and light chains of palivizumab. The final product presented about 70-fold higher affinity for the F protein of RSV and 20-fold more potency than palivizumab [94]. In clinical trials, also conducted in infants, motavizumab showed a pharmacokinetic profile similar to palivizumab [94]. A Phase 2 study was conducted in infants randomly assigned to receive monthly intramuscular injections of motavizumab or palivizumab, demonstrating similar results in overall adverse events (AEs) rates and development of antidrug antibodies [95]. Even when tested in children with hemodynamically significant CHD, motavizumab resulted comparable to palivizumab [96]. Despite these promising results, in a phase 3, randomized, double-blind, palivizumab-controlled study, motavizumab recipients developed a large number of AEs, mostly cutaneous, and the FDA did not authorize the commercialization of this drug [30,84]. The substitutions of three amino acids (M252Y/S254T/T256E, YTE) created an empowered form of motavizumab, called mota-YTE, that showed an extended half-life up to 100 days (4-fold longer than Motavizumab and Palivizumab) in healthy adult volunteers [85]. These results support the application of YTE technology to reduce the dosing frequency for RSV prevention. Nevertheless, further development of mota-YTE has not been continued after concerns of FDA on motavizumab [36]. Suptavumab, also known as REGN2222, was another IgG_1_ mAb against the RSV F glycoprotein. After demonstrating in vitro that it was 36-fold more potent than palivizumab, suptavumab showed a comparable safety with placebo in healthy adult volunteers [97]. More recently, a Phase 3 trial was conducted on 1154 preterm infants who were ineligible or without access to palivizumab. Patients were treated with 1 or 2 doses (administered 8 weeks apart) of intramuscular suptavumab (30 mg/kg bw). No significant differences were observed for RSV-related hospitalization or outpatient LRTI rates between placebo and suptavumab, probably because of a new circulating mutant strain of RSV B unresponsive to this mAb [86]. The presence of two amino acid mutations in the suptavumab epitope found on all circulating RSV-B strains, in fact, rendered this mAb unable to bind and neutralize them. In August 2017, the Sponsor Agency announced that the trial failed to achieve its primary efficacy endpoint of prevent serious RSV-related LRTI, so suptavumab was discontinued [98]. RB1 is an entirely human mAb IgG_1_ against the antigenic site IV of the RSV F protein. This site, together with the antigenic site III, results highly conserved across all RSV genotypes. The advantage of a mAb that binds these specific epitopes consists in the neutralization and consequent protection from diverse RSV A and B strains. Preclinical studies demonstrated a potent in vivo protection in cotton rats [99,100]. MK-1654 can be considered an improved version of RB1. It is the same mAb with a YTE aminoacidic chains’ substitution (M252Y/S254T/T256E) that extends its half-life. Currently, MK-1654 is the object of clinical trials. In a double-blinded, Phase 1 study involving 152 healthy adult volunteers, it resulted safe as the placebo and its half-life amounted to an average of 73–88 days [100]. A Phase 2a trial enrolled 80 healthy adults to determine if a single intravenous dose of MK-1654, when administered at one of four dose levels, decreases viral RSV load compared to placebo. Completed in August 2020, the results of this trial are not available yet [92]. The first trial focused on infants started in September 2018 and is still recruiting. The aim of this study is to evaluate the safety and tolerability of a single ascending doses of MK-1654 in a total of 180 healthy preterm (29–35 wGA) and full-term (>35 wGA) infants that will be checked up to 545 days from the mAb injection [93]. 

Recently, research focused on the prefusion form of viral F protein (called pre-F protein) that differs from the proteic form after viral fusion with the host cell. In the prefusion form, F protein exhibits epitopes that result highly conserved in different RSV serotypes. MEDI-8897 (also called nirsevimab) is an example of this type of mAbs that could represent a real improvement in passive immunoprophylaxis for RSV. Nirsevimab is an IgG_1_ mAb that targets antigenic site ∅ on the F glycoprotein of RSV, demonstrating a greater neutralizing potency than palivizumab. Moreover, its half-life results extended thanks to YTE technology (the triple amino acid substitutions also used for mota-YTE), reaching the possibility of protection against RSV for an entire season with a single administration [101]. Nirsevimab showed a mean half-life of about 80–120 days and a favorable safety profile in randomized, double-blind, placebo-controlled clinical trials conducted in healthy adults and healthy preterm infants [102,103]. In a multicenter randomized placebo-controlled trial conducted in premature infants, nirsevimab reduced the risk of RSV-LRTI (absolute risk reduction (ARR) 6.9%; number needed to treat (NNT) 14.5) and hospitalization (ARR 3.3%; NNT 30.3) respectively [87,88]. A Phase 3 study is currently recruiting about 3000 healthy late preterm and term infants to determine the efficacy of niservimab in these patients, who would not be eligible to receive RSV prophylaxis [89]. In July 2019 another trial started directly comparing nirsevimab to palivizumab in high-risk children, whereas in August 2020 this mAb started to be tested in immunocompromised Japanese children aged ≤2 years [90,91]. Recruitment for these studies is still open. 

Some RSV mAbs have been tested only in preclinical trials; their main results are summarized in Table 3. 

To date, four mAbs against RSV pre-F protein have been evaluated in preclinical studies. Pre-F protein exhibits epitopes that result highly conserved not only in different RSV serotypes, but even in human metapneumovirus (hMPV), a member of the *Pneumoviridae* family, which also includes RSV. The advantage of mAbs that target pre-F protein epitopes consists in efficacy against both these viral agents (RSV and hMPV) that are responsible for a great number of LRTI in infants. The first IgG_1_ mAb against pre-F protein which showed prophylactic and therapeutic efficacy against both RSV and hMPV in mice was MPE8 [104]. Also, 54G10 was able to neutralize both hMPV and RSV activity in cell cultures and showed efficacy against these viruses in mouse models [105]. 25P13 was a mAb identified from blood donors that showed a similar binding profile to MPE8 and consequently the same efficacy in cross-reactivity against both RSV and hMPV [106]. Another mAb, called 101F and binding to the antigenic site IV of RSV F, was initially found to cross-react with MPV postfusion F and to neutralize both RSV and hMPV, but in a more recent in vivo study, no detectable cross-reactivity was noticed in mice [111]. Recently, the 17E10 was analyzed, and it showed a binding pose similar to 101F, with in vitro cross-reactivity against both RSV and hMPV [107]. To date, no studies involving humans are available for this type of mAbs. 

The viral G protein also represented a target for mAbs. Unfortunately, only a small part of this protein is well-conserved in both RSV A and B strains, making it hard to obtain mAbs with efficacy against both serotypes. Nevertheless, the G protein is implicated in the pathogenesis of RSV disease because it controls the virus-induced immune response and lung inflammation, and its blockage represents a real strength for projected mAbs [36]. To date, four mAbs against RSV G glycoprotein have been evaluated in preclinical trials. In vivo studies involving the anti-RSV G protein mAb 131-2G demonstrated decreased levels of RSV loads and lung inflammation when this product was administered in a precocious phase of the disease in mice [112,113]. The mAb 131-2G has been shown to prevent the viral G protein from binding to CX3CR1, a receptor located in human airway epithelial cells. It consequently inhibits the host infection and the RSV disease [36]. In previously RSV-infected mice, 131-2G administration appeared to significantly reduce bronchoalveolar lavage inflammatory cells number, airway reactivity (measured by pulse oximeter), and cytokines of T helper type 2 lymphocytes more rapidly than 143-6C, a mAb against RSV F protein similar to palivizumab. These results were associated with both antiviral and anti-inflammatory activity of 131-2G [108]. Similar results emerged from an in vivo study focusing on 2B11 and 3D3, two mAbs that bind epitopes similar to 131-2G, and whose comparison with palivizumab and normal human IgG administration at prophylactic and therapeutic dosages showed a significant decrease in inflammatory cells and cytokines [109]. Another mAb against the RSV surface G glycoproteins (GD-mAb) effectively neutralized RSV in vitro and significantly reduced the lung viral load in mice [110]. No clinical trials on this type of mAbs are currently available for humans. 

## 3. Conclusions

Since the documented risk of adverse events associated with RSV-IGIV, mAbs have been the milestones of passive RSV prophylaxis. Currently, the only licensed mAb is palivizumab, approved by the FDA in 1998. Given its high cost, palivizumab is recommended only in high-risk infants, including those born preterm and those with BPD and hemodynamically significant CHD, and in selected cases of clinical rare pathologic conditions (i.e., neuromuscular disease, congenital anatomic pulmonary abnormalities, severe immunodeficiency). However, many pediatric RSV-related hospitalizations do not fully meet the criteria for palivizumab and, in developing countries, palivizumab is not available. For these reasons, new mAbs with a potential better cost-effectiveness ratio than palivizumab are under development. Some of them, like motavizumab and suptavumab, have failed over the years for different reasons. Long-lasting mAbs with strong neutralizing activity like niservimab are the most promising candidates, as they might lead to protection of infants for an entire RSV season with a single administration. 

## Figures and Tables

**Table 1:** Comparison of the 2009 and 2014 AAP guidelines on RSV prophylaxis with palivizumab.

Patient Group	2009 Recommendations [54]	2014 Recommendations [13]
Preterm infants	• Infants born <32 WGA • Infants from 32 to 35 WGA with at least 1 of the following risk factors: 1. Attending childcare; 2. Living together with siblings or other children younger than 5 years	• Infants born <29 WGA who are <12 months at the start of the RSV season
BDP	• Children <24 months with BLD who receive medical therapy within 6 months before the start of the RSV season • Patients with the most severe BLD continuing to require medical therapy may benefit from prophylaxis during a second RSV season	• Infants with BLD born <32 WGA requiring oxygen therapy for at least the first 28 days of life, in the first year during the RSV season; in the second year only if they continue to require medical support during the 6 months before the start of RSV season
CHD	• Children younger than 24 months of life with haemodynamically significant cyanotic or acyanotic CHD	• Certain children younger than 12 months of life with haemodynamically significant CHD
Anatomic pulmonary abnormalities or neuromuscular disorder	• Infants who have either significant congenital abnormalities of the airway or a neuromuscular condition that compromises respiratory tract secretions management	• Infants with neuromuscular disease or congenital anatomic pulmonary abnormalities that alter the clearence of secretions in the airways because of ineffective cough
Immuno-compromised	• Specific recommendations cannot be made, but infants with CHD and severe immunodeficiency may benefit from prophylaxis	• Children <24 months who are profoundly immunocompromised during the RSV season
Down Syndrome	• No recommendation	• Not recommended in children with Down syndrome unless other risk factors are present
Cystic fibrosis	• No recommendation	• Infants with cystic fibrosis with evidence of BPD and/or nutritional compromise
Breakthrough RSV hospitalization	• If an infant who is receiving palivizumab experiences an RSV infection, prophylaxis should continue	• If any infant receiving palivizumab experiences an RSV hospitalization, monthly prophylaxis should be discontinued

BPD = bronchopulmonary disease, bw = body weight, CHD = congenital heart disease, RSV = Respiratory Syncytial Virus.

**Table 2:** Overview of the RSV mAbs in clinical development; only the most recent and the ongoing trials are reported.

mAb	Target Site	RCT’s Characteristics and Phase, Enrollment’s Time and Cohort Dosage and Route Published Result Summary	Study ID Ref.
MEDI-493 (Palivizumab)	RSV F glycoprotein (IgG_1_)	Multicenter, randomized, placebo-controlled, phase 3 trial enrolling 1502 premature or BPD infant Monthly i.m. administration of MEDI-493 (15 mg/kg bw) Comparing palivizumab prophylaxis vs. placebo: global reduced incidence of RSV-related hospitalization by 55% (hospitalization in palivizumab group 4.8% vs. placebo group 10.6%, *p* = 0.00004) reduced incidence in premature infants without BPD by 78% and in BPD infants by 39% No significant differences in reported adverse events between the two groups	[29]
RSHZ19 (SB 209763)	RSV F glycoprotein (IgG_1_)	Phase 3 trial with 800 recruited at-risk infants (unknown enrollment’s time) Monthly/bimonthly i.m. administration of RSHZ19 (10 mg/kg bw): failure in protection against RSV disease	(D. Burch, personal communication) cited in [45]
HNK20	RSV F glycoprotein (IgA)	Multicenter controlled trial (NA phase) conducted on more than 600 at-risk infants for severe RSV i.n. administration of HNK20 (NA timing and dosage) vs. placebo: treatment with HNK20 did not result in a significant decrease in the incidence of hospitalization for RSV LRTI	Cited in [83]
MEDI-524 (Motavizumab)	RSV F glycoprotein (IgG_1_)	Multicenter, double-blind, randomized, non-inferiority, palivizumab-controlled, phase 3 trial enrolling 6635 preterm or with CLD infants, with monthly i.m. administration of motavizumab or palivizumab (15 mg/kg bw): Relative reduction in RSV hospitalization by 26% (achieving non-inferiority to palivizumab) Overall, no significant difference of reported AE between groups Cutaneous events reported in 2 percentage points more in motavizumab than palivizumab group (7.2% vs. 5.1%)	ClinicalTrials.gov registration number NCT00129766 [84]
Motavizumab-YTE	RSV F glycoprotein (IgG_1_ with M252Y/S254T/T256E amino acidic substitution)	Double-blind, randomized, placebo-controlled, single-dose, escalation study (phase 1) enrolling 31 healthy adults, randomized to receive a single i.v. dose of motavizumab-YTE or motavizumab (0.3, 3, 15, or 30 mg/kg) and followed for 240 days: Significantly lower clearance of motavizumab-YTE than motavizumab (71% vs. 86%) 2- to 4-fold longer half-life (t1/2) of motavizumab-YTE than motavizumab Comparable safety and incidence of ADA between motavizumab-YTE and motavizumab	ClinicalTrials.gov registration number NCT00578682 [85]
REGN-2222 (Suptavumab)	RSV F glycoprotein (IgG_1_)	Double-blind, randomized, placebo-controlled, phase 3 trial enrolling 1154 preterm infants ineligible or without access to palivizumab over 3 RSV seasons (November 2015–September 2017), i.m. suptavumab (30 mg/kg bw, 1 or 2 doses administered 8 weeks apart) vs. placebo: no significant differences between primary endpoint (RSV-related hospitalization or outpatient LRTI) rates (8.1%, placebo vs. 7.7%, 1-dose vs. 9.3%, 2-dose) failure in reducing RSV hospitalizations or outpatient LRTI because of a newly circulating mutant strain of RSV B	ClinicalTrials.gov registration number NCT02325791 [86]
MEDI-8897 (Nirsevimab)	site Ø of the prefusion conformation of F glycoprotein (IgG_1_ with YTE amino acidic substitution)	Multicenter, double-blind, randomized, placebo-controlled, phase 2b trial enrolling 1453 preterm infants between Nov 2016 to Nov 2017 im administration of a single dose of nirsevimab (50 mg) vs. placebo and follow-up for 360 days: mean half-life 59.3 ± 9.6 days similar ADA responses (MEDI-8897 5.6% vs. placebo 3.8%) nirsevimab reduced RSV-LRTI compared to placebo (ARR 6.9%; 95% CI, 4.1%–9.7% and NNT 14.5; 95% CI, 10.3–24.3) nirsevimab reduced hospitalization for RSV-disease compared to placebo (ARR 3.3%; 95% CI, 1.4%–5.2%; NNT 30.3; 95% CI, 19.4–69.5)	ClinicalTrials.gov registration number NCT02878330 [87,88]
		Multicenter, double-blind, randomized, placebo-controlled, phase 3 trial recruiting 3000 healthy late preterm and term infants not eligible to receive palivizumab’s prophylaxis, the study started in July 2019 and it is still in progress (estimated completion date in April 2023), NA dosage and timing of nirsevimab, Follow-up for 510 days after dosing: Primary outcome (incidence of medically attended LRTI due to RT-PCR confirmed RSV 150 days post-administration): results NA yet	ClinicalTrials.gov registration number NCT03979313 [89]
		Multicenter, double-blind, randomized, Palivizumab-controlled, phase 2/3 study enrolling 1500 high-risk children (preterm infants without CLD/CHD or infants with CLD or with hemodynamically significant CHD), the trial is recruiting since July 2019 (estimated completion date in December 2021) Primary outcome (safety and tolerability of nirsevimab): results NA yet	ClinicalTrials.gov registration number NCT03959488 [90]
		Open-label, uncontrolled, single-dose study enrolling 30 immunocompromised Japanese children aged <2 years since Aug 2020 for 2 RSV epidemic seasons (estimated completion date in Nov 2022), i.m. administration of nirsevimab (50 mg if bw < 5 kg or 100 mg if bw ≥ 5 kg if patients are enrolled during their 1st RSV season, whereas subjects entering their 2nd RSV season will receive a single fixed 200 mg dose), and follow-up for 1 year Primary outcome (safety, tolerability, occurrence of ADA, and efficacy of nirsevimab): results NA yet	ClinicalTrials.gov registration number NCT04484935 [91]
MK-1654	site III of theF glycoprotein (IgG_1_ with YTE amino acidic substitution)	Double-Blind, randomized, placebo-Controlled, phase 2a study enrolling 80 healthy adults, Oct 2019–Mar 2020 (estimated study completion date: Aug 2020), with a single i.v. administration of MK-1654 (4 different dose levels, NA dosage for each level) and subsequent i.n. inoculation with RSV Primary outcome (VL-AUC from day 2 through day 11, and from day 31 through day 40 after viral inoculation): results NA yet	ClinicalTrials.gov registration number NCT04086472 [92]
		Double-blind, randomized, placebo-controlled, single ascending dose, phase 1/2, recruiting 180 healthy preterm and full-term infants since Sep 2018 (estimated study completion date: Oct 2021), i.m. administration of MK-1654 (randomization into 1 of 4 dose escalation groups) and follow-up for 545 days Primary outcome (safety, tolerability, pharmacokinetics, and incidence of ADA): results NA yet	ClinicalTrials.gov registration number NCT03524118 [93]

ADA = antidrug antibody, AE = Adverse Event, ARR = absolute risk reduction, BPD = bronchopulmonary disease, bw = body weight, CHD = congenital heart disease, CI = confidence interval, CLD = Chronic Lung Disease, HR = hazard ratio, i.m. = intramuscular, i.n. = intranasal, IQR = interquartile range, i.v. = intravenous, LRTI = Lower Respiratory Tract Infection, NA = not available, NNT = number needed to treat, RSV = Respiratory Syncytial Virus, RT-PCR = Real Time–Polymerase Chain Reaction, VL-AUC = Area Under the Viral Load-time Curve.

**Table 3:** Summary of the RSV mAbs in preclinical development; only the most recent trials are reported.

mAb	Target Site	Characteristics of In Vitro or In Vivo Study, Dosage and Route, Published Result Summary	Ref.
MPE8	Prefusion form of F glycoprotein (2 highly conserved anti-parallel b-strands)	In vivo study conducted on 6–8-week-old female of BALB/c mice or 129S6/svEv-Stat1-deficient mice, with i.v. administration of MPE8 at different doses (varying from 0.12 to 30 mg/kg bw), MPE8 showed potent prophylactic efficacy of hRSV and hMPV infection, and both a prophylactic and a therapeutic effect against pneumonia virus of mice	[104]
54G10	Prefusion form of F glycoprotein	In vitro analyses were conducted on LLC-MK2 cells and Hep-2 cells In vivo study was conducted on 6-week-old female DBA/2 (permissive for all 4 hMPV subgroups) and BALB/c mice, with administration of 54G10 at different doses (0.2 mg/Kg and 0.6 mg/Kg, NA route): 54G10 neutralized all 4 subgroups of hMPV in vitro and it was both prophylactic and therapeutic against hMPV in vivo 54G10 also in vitro neutralized RSV activity and it was both prophylactic and therapeutic against hRSV in vivo	[105]
25P13	Prefusion form of F glycoprotein (identified Ab from a blood donor with conserved surface patch of residues similar to MPE8)	In vitro 25P13 showed to target the same conserved surface patch of residues on F similar to MPE8 (HCDR1 and HCDR2 are >80% conserved)	[106]
17E10	Prefusion form of F glycoprotein (antigen IV site)	In vitro analyses were conducted on Hep-2 cells and Vero cells: 17E10 showed a binding pose similar to the mAb 101F, results suggested that binding to the antigenic site IV is indicative of cross-reactivity with hMPV and hRSV	[107]
131-2G	G glycoprotein	In vivo study was conducted on 6-week-old female of BALB/c mice, with RSV inoculation (10^6^ TCID_50_/50 µL) on day 0, administration of 131-2G (anti-G protein) or 143-6C (anti-F protein similar to palivizumab) (300 µg/mL) or nothing on day 3, lung and BAL collection on days 4–8, pulse oximeter measurements on days 6, 8, 10 and 12 131-2G associated with a rapid decrease in the total BAL inflammatory cell number compared to untreated mice for all days after treatment (*p* < 0.05), differently from treatment with 143-6C compared to untreated mice (*p* ≥ 0.05) 131-2G associated with decreased airway dysfunction measured by pulse oximeter (*p* ≤ 0.001) at all time points, differently from 143-6C, associated with a decrease only since day 8	[108]
2B11 and 3D3	G glycoprotein	In vivo study was conducted on 4–6-week-old, specific-pathogen-free, female BALB/c;different groups with i.p. administration of of 2B11, 3D3, palivizumab, or normal human IgG;prophylactic treatment: administration 1 day prior to i.n. RSV infection of different dose levels(5 mg/kg, 1.5 mg/kg, 0.15 mg/kg, 0.015 mg/kg or 0.0015 mg/kg);therapeutic treatment: administration on day 3 post-i.n. RSV infection of 5 mg/kg bw Prophylactic treatment: significantly reduced BAL CD3+ (only 2B11), CD11b+, B220+, and DX5+ (2B11 and 3D3), NK cells (2B11 and 3D3) comparing to palivizumab and normal human IgG Therapeutic treatment: decreased viral lung titers at day post-inoculation (2B11, 3D3, but also palivizumab), reduction in total lung leukocytes on day 5 (2B11, 3D3) differently from palivizumab, that did not reduce total BAL cells on day 5	[109]
GD-mAb	G glycoprotein	In vitro analyses were conducted on Vero cells In vivo study was conducted on 8-week-old female of BALB/c mice, different groups with administration of GD-mAb (different dose levels: 3 mg/Kg, 1.5 mg/Kg, 0.32 mg/Kg, 0.16 mg/Kg, 0.032 mg/Kg) vs. ribavirin (0.05 g/kg per day) vs. nothing In vitro: GD-mAb associated with RSV neutralization In vivo: GD-mAb significantly decreased the viral titer in the lungs and the pulmonary inflammatory response	[110]

BAL = bronchoalveolar lavage, BALB = Bagg and albino, h = human, HCDR = high complementarity-determining region, i.n. = intranasal, i.p. = intraperitoneal, hMPV = human Metapneumovirus, hRSV = human Respiratory Syncytial Virus.

## Data Availability

Not applicable.
